# Purification of Drinking Water by Filtration

**Published:** 1896-04

**Authors:** 


### Purification of Drinking-Water by Filtration.

We reproduce the following from the *New York Medical Record* as being timely and important, as the pollution of our streams everywhere is a serious consideration as affecting the public health. It will be read with interest:

"The importance of pure water in determining the health of a community has long been recognized and can not be overestimated.

"At the present time, it is impossible for many cities and large towns to obtain the required amount of water from a naturally pure source, and in the future, with the enormous increase in population and the number of manufacturing towns established along the banks of the small streams and rivers, this difficulty will be manifestly greater. Therefore the possibility of purifying by artificial means water which has been polluted by sewage, and which contains both organic matter and bacteria, has become a question of great importance in many communities.

"In considering any method for the accomplishment of this object, two things must be borne in mind, viz., its efficiency and its cost. The objections which have been urged against filtration are:

First, that while a filter might remove the coarse material in suspension, it it would allow all the organic matter in solution, and the bacteria, to pass through unchanged.

"Second, that even if a filter were efficient for a short time, it soon becomes clogged and saturated, and then the condition of water which passes through is worse than when it entered.

"Third, that the cost and maintenance of a properly constructed filter is so great that it can not be universally adopted as a means of purifying water.

"The report of the Massachusetts State Board of Health for the year 1894, contains some very interesting and important facts upon all these points.

"For the past seven years the board has maintained an experimental station at Lawrence, for the sole and express purpose of testing the efficacy of filtration of water to purify it and render it fit for household purposes. The water tested was that of the Merrimac river, which is lined from source to mouth with manufacturing towns, and which may be taken as a fair sample of river water contaminated with a considerable amount of organic matter.

The filters were of all sizes and thicknesses, from those of a few feet square and ten inches in depth, to the large filter covering two and one-half acres, through which the water supplied to the city of Lawrence has been filtered since 1893.

"Chemical and bacteriological examinations were made weekly, and sometimes daily, of the water of ingress and egress. Sand of different sizes was used, and the filters were run both intermittently and continuously.

"The results of this careful and painstaking investigation, extending over a number of years, and every source of error being eliminated, are both astonishing and gratifying.

"From a bacteriological standpoint they prove that a properly constructed and properly managed filter will remove from 98 to 99.84 per cent. of the ordinary bacteria in water, and that if such bacteria as the bacillus prodigiosus, which is very similar to the typhoid bacillus, be added to the water in varying proportions, the filter will remove from 99 to 99.93 per cent. The organic matter in solution is greatly diminished and the water is chemically purified.

"Moreover, the efficiency of the filter instead of diminishing increases with age and use, owing to the formation of a gelatinous coating.about each grain of sand, which serves to entangle the bacteria iu their progress.

"The rate of filtration may reach five million gallons daily per acre of filter without impairing the efficiency. If the surface clogging is properly removed, there will be no appreciable difference in the quality of the filtered water during or after the process of removal.

"Finally, the cost of construction and maintenance of such filters is not so great as was supposed, and is not to be compared with the benefits derived from their use. The one which has been in successful use in the city of Lawrence proves that the plan is practicable in supplying cities with potable water. It seems to us that the knowledge derived from these experiments should be spread abroad and the attention of municipal authorities called to them.

"In the immediate vicinity of New York the water of the Passaic River has been for a long time very bad, and is constantly growing worse as regards its contamination with sewage and the waste of manufacturing plants on its banks. Several large cities and towns obtain their water supply from this source, and if there is a practical and economical means of rendering this water pure and wholesome, it certainly ought to be adopted.

"While our own Croton is considered a very good quality of water, yet it is liable to contamination, and we have recently had abundant proof that it may become disagreeable to sight and taste. A proper system of filtering would no doubt improve the quality greatly, and the matter should be brought to the attention of the proper authority."—*Ed. N. Y. Medical Record.*

